# The efficacy of remote ischemic conditioning in improving neurological function and short-term prognosis in acute ischemic stroke: a prospective controlled study

**DOI:** 10.3389/fneur.2025.1542833

**Published:** 2025-07-01

**Authors:** Peiqi Huang, Kun Lin, Liling Wei, Qiong Zeng

**Affiliations:** ^1^Department of Rehabilitation, The First Affiliated Hospital of Shantou University Medical College, Shantou, Guangdong Province, China; ^2^Department of Endocrinology, The First Affiliated Hospital of Shantou University Medical College, Shantou, Guangdong Province, China; ^3^Department of Neurology, The First Affiliated Hospital of Shantou University Medical College, Shantou, Guangdong Province, China

**Keywords:** remote ischemic conditioning, ischemic stroke, modified Rankin scale scores, national institutes of health stroke scale, activities of daily living scores

## Abstract

**Objective:**

To evaluate the efficacy of remote ischemic conditioning (RIC) in improving neurological function and short-term prognosis in patients with acute ischemic stroke (AIS).

**Methods:**

This randomized, controlled, single-blind study aimed to evaluate the short-term (7-day) effects of RIC on neurological function in patients with AIS. 264 AIS patients (median age 65 years, 63.3% male) with ischemic symptoms <72 h post-onset were randomly assigned to either the RIC group (*n* = 65) or the control group (*n* = 199). RIC was administered manually using a cuff sphygmomanometer, while the control group received a sham RIC treatment. Patients with cardioembolic sources or a history of prior stroke were excluded from the study. Primary outcomes were the proportion of patients with an mRS score of ≤2 at 7 days, as well as changes in the National Institutes of Health Stroke Scale (NIHSS), modified Rankin Scale (mRS), Activities of Daily Living (ADL), and Rancho Los Amigos (RLA) scores. Data were collected at baseline and 7 days post-enrollment, with in-person follow-up visits conducted by blinded clinicians.

**Results:**

At 7 days, the RIC group showed a significantly higher proportion of patients with an mRS score of ≤2 compared to the control group (41.5% vs. 28.1%, *p* = 0.043). Significant improvements were observed in the RIC group compared to the control group in NIHSS (*p* = 0.004) and ADL scores (*p* = 0.005), but not in RLA scores (*p* > 0.05). Binary Logistic Regression Analysis indicated that, after adjusting for baseline factors, the treatment effect of RIC remained statistically significant.

**Conclusion:**

RIC treatment enhances neurological function and improves short-term prognosis in AIS patients. These findings support the potential clinical application of RIC in AIS management.

## Introduction

Acute ischemic stroke (AIS) is one of the leading causes of disability and mortality worldwide (1). Despite significant advancements in reperfusion therapies, such as intravenous thrombolysis and mechanical thrombectomy, these interventions remain limited by narrow therapeutic windows and are accessible to only a fraction of patients (2, 3). Even among those who receive timely treatment, the rates of mortality and long-term disability remain alarmingly high, necessitating additional strategies to improve clinical outcomes (4).

Remote ischemic conditioning (RIC), initially introduced for myocardial protection, has gained attention for its potential neuroprotective effects in AIS. RIC involves inducing brief episodes of ischemia and reperfusion in distant tissues, such as limbs, to trigger protective systemic responses that may mitigate ischemic injury in the brain (5, 6). The underlying mechanisms are believed to include the reduction of oxidative stress, suppression of inflammation, stabilization of the blood–brain barrier, and enhancement of collateral circulation (7, 8). However, clinical studies on RIC in stroke have produced mixed results, possibly due to variations in treatment protocols, patient selection, and timing of the intervention (9–12).

Notably, the RICAMIS study demonstrated the efficacy of repeated remote ischemic postconditioning in improving outcomes for AIS patients, providing significant evidence of RIC’s potential in stroke treatment (13). This highlights the importance of optimizing RIC protocols and identifying the patient subgroups most likely to benefit from this intervention. This study aims to assess the efficacy of RIC in improving neurological function and short-term prognosis in AIS patients. Additionally, we explore independent predictors of treatment outcomes. By addressing these gaps, we hope to provide insights that could guide the future clinical application of RIC in AIS management.

## Materials and methods

### Study design and subjects

This single-center, prospective, interventional controlled study recruited 266 AIS patients admitted to the Neurology Department of the First Affiliated Hospital of Shantou University Medical College from April 2021 to November 2022. Patients were randomly assigned to either the RIC group or the control group using a 1:3 allocation ratio. This method was employed to optimize resource allocation while maintaining statistical power, as preliminary data suggested greater variability in outcomes within the control group. The randomization process was conducted using a computer-generated randomization list, ensuring an unbiased assignment of patients to each group. The allocation was concealed from the study personnel and patients to maintain blinding, and group assignment was revealed only after the patient’s enrollment. Based on preliminary data and prior studies, the significant efficacy rate (defined as mRS ≤ 2) was estimated to be 60% in the RIC group and 30% in the control group. With a significance level of *α* = 0.05 (two-tailed), a power of 90% (*β* = 0.10), and an estimated consistency rate of 35% between the two groups, the sample size was calculated as follows: $n=\frac{((Z_{\alpha }+Z_{\beta })\cdot \sqrt{2\cdot \bar{p}\cdot (1-\bar{p})}+Z_{\alpha }\cdot \sqrt{p_{1}\cdot (1-p_{1})+p_{2}\cdot (1-p_{2})})2}{(p_{1}-p_{2})2}$ The calculated sample size for the total study was 144 participants, with 36 participants in the RIC group and 108 participants in the control group, based on the 1:3 allocation ratio. Due to practical recruitment constraints and baseline variability, the study ultimately included 67 RIC and 199 control participants, ensuring robust statistical power.

Inclusion criteria were: ① meeting the AIS diagnostic criteria (14); ② having ischemic symptoms within 72 h before admission, with CT or MRI excluding cerebral hemorrhage and imaging showing an infarct corresponding to the abnormal brain function location; ③ age between 18 and 85 years. Exclusion criteria were: ① cardiogenic cerebral embolism. Patients with stroke of cardioembolic origin, including those with atrial fibrillation, recent myocardial infarction, or other heart conditions that increase the risk of cardioembolic stroke, were excluded. The rationale for excluding these patients is that cardioembolic strokes have a distinct pathophysiology and treatment strategy compared to other ischemic stroke subtypes. The effects of RIC may differ in this subgroup due to the involvement of systemic emboli, and including such patients could introduce significant confounding factors in assessing the efficacy of RIC.; ② history of cerebral infarction, thrombolysis within the past month, or transient ischemic attack; ③ severe heart, lung, liver, kidney, or hematological diseases (e.g., heart, liver, kidney failure), or malignancy; ④ upper limb soft tissue and vascular injury, limb deformity, arteriovenous thrombosis, or systolic blood pressure exceeding 200 mmHg; ⑤ lower limb soft tissue or vascular injury, peripheral vascular disease, or limb deformity; ⑥ Reperfusion Therapies (IV Thrombolysis and/or Thrombectomy). This study was conducted in accordance with the CONSORT guidelines for randomized controlled trials. It was prospectively registered with the Chinese Clinical Trial Registry (ChiCTR2100042225). The study was approved by the ethics committee of the First Affiliated Hospital of Shantou University Medical College (Ethical Approval Number: B-2020-174). All participants had signed the informed consent.

### RIC intervention

The RIC intervention used in this study is based on remote ischemic postconditioning. This strategy involves the application of intermittent ischemia and reperfusion to a limb (usually the arm or leg) to induce systemic protective effects. The protocol followed in this study adheres to established RIC procedures commonly used for neuroprotection in AIS patients. All patients were hospitalized during the treatment period. The RIC intervention was administered by trained healthcare professionals. The RIC intervention involved using a cuff sphygmomanometer (model RIP-908S, Shenzhen Lizhongsong Industrial Co., Ltd.) to inflate and compress the brachial/femoral artery unilaterally for 5 min at a pressure of 200 mm Hg, followed by 5 min of deflation. This process was repeated on the other limb in the same manner for five cycles. Patients in the intervention group underwent RIC treatment once daily for 1 week after admission. The control group received sham RIC treatment, which was similar to RIC treatment, except the cuff pressure was set at 30 mm Hg. Adverse events were monitored daily during hospitalization by trained clinical neurologists using standardized adverse event case report forms. Predefined safety indicators included hemorrhagic cerebrovascular events, limb pain or subcutaneous hemorrhage during RIC, seizures, pneumonia, deep vein thrombosis, pulmonary embolism, and myocardial infarction. All events were classified according to severity (mild, moderate, severe) and causality (related, possibly related, unrelated). No adverse events were reported in either the RIC or control group during the study period.

### Clinical measures

In this study, the key variables analyzed included stroke severity, neurological function, and cognitive function. Stroke severity was assessed using the National Institutes of Health Stroke Scale (NIHSS), a well-established measure of neurological deficit. Functional outcomes were measured using the Modified Rankin Scale (mRS) and Activities of Daily Living (ADL) scores. Cognitive function was assessed using the Rancho Los Amigos Cognitive Scale (RLA), which evaluates the degree of cognitive recovery post-stroke. Data were collected at baseline (Day 0) and 7 days post-enrollment. For all patients, in-person follow-up visits were conducted to assess the primary and secondary outcomes. During these visits, trained clinicians administered the NIHSS, mRS, ADL, and RLA assessments. All follow-up assessments were performed by individuals who were blinded to the treatment group. Cerebral infarction was classified using the TOAST criteria. Blood samples were collected on the first day and the seventh day for inflammation markers (white blood cell count, C-reactive protein) and coagulation function indicators (prothrombin time, activated partial thromboplastin time, fibrinogen).

Primary Objective: The primary endpoint is the proportion of patients with a modified Rankin Scale (mRS) score of ≤2 at day 7.

Secondary Objective: To evaluate additional functional outcomes such as changes in NIHSS, ADL and RLA scores.

### Statistical analyses

Statistical analyses were performed using SPSS 27.0. The Shapiro–Wilk test was used to assess whether continuous variables were normally distributed. Variables with a normal distribution are presented as mean ± standard deviation; between-group differences were evaluated by independent-samples t-test and within-group differences by paired-samples t-test. Variables with a non-normal distribution are reported as median (interquartile range) and compared between groups using the Mann–Whitney U test; within-group comparisons were made with the Wilcoxon signed-rank test. Ordinal variables were also analyzed using the Mann–Whitney U test. Categorical variables are expressed as counts (percentages) and compared using the χ^2^ test or Fisher’s exact test, as appropriate. Finally, binary logistic regression was applied to examine the relationship between different treatment modalities and post-treatment outcomes. The prognostic outcome (mRS on day 7 ≤ 2 as the good prognosis group, assigned a value of 0; mRS on day 7 > 2 as the poor prognosis group, assigned a value of 1) was used as the dependent variable. Logistic analysis involved 3 models: Model 1: unadjusted model. Model 2: Adjusted for baseline characteristics such as age, sex, and baseline mRS scores (day 0). Model 3: Further adjusted for additional variables that might influence outcomes, such as hyperlipidemia and HbA1C levels. A *p*-value<0.05 was considered statistically significant.

## Results

### Baseline characteristics

**Figure 1:**
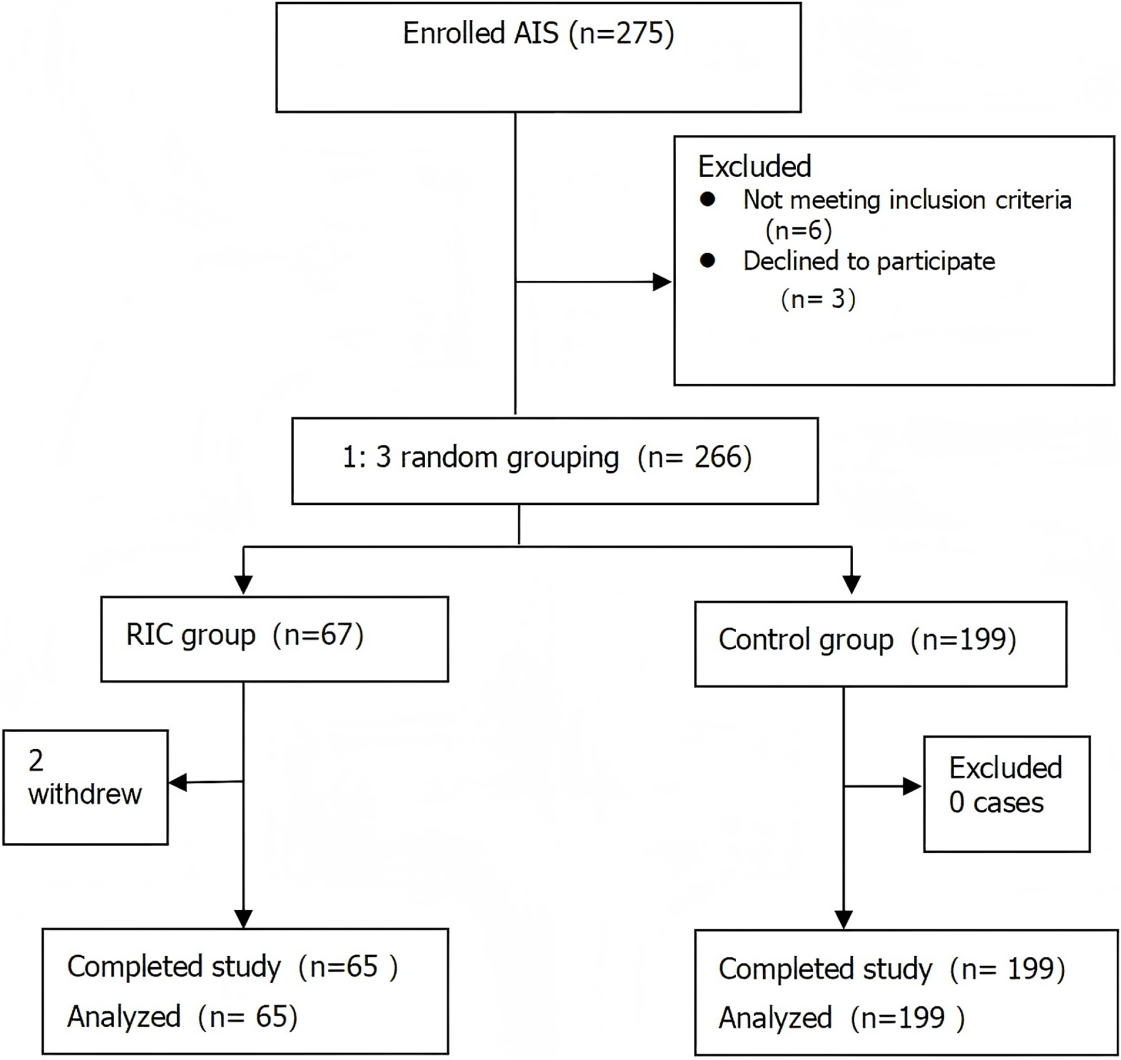
Trial flow. RIC indicates remote ischemic conditioning.

Two participants from the RIC group withdrew from the study midway, leading to a final count of 65 participants in the RIC group and 199 in the control group (Figure 1). In total, 264 participants completed the study, with a median age of 65 years, 63.26% male, and an average onset time of 29 h for cerebral infarction. There were statistically significant differences between the intervention and control groups in terms of hyperlipidemia prevalence and HbA1C levels (*p* < 0.05), while other variables such as age, gender, and stroke severity were not significantly different (*p* > 0.05; Table 1).

**Table 1:** Analysis of clinical data of patients in RIC group and control group.

Variable	Control group (*n* = 199)	RIC group (*n* = 65)	Z/t/*χ^2^*	*p* value
Demographic characteristics				
Age (years)	66.00 (58.00,73.00)	65.00 (56.50,71.00)	−1.335	0.182
Gender (Male %)	127 (63.8)	40 (61.5)	0.110	0.741
Comorbidities and Risk Factors				
Hypertension (%)	167 (83.9)	54 (83.1)	0.026	0.873
Diabetes (%)	120 (60.3)	31 (47.7)	3.182	0.074
Coronary Heart Disease (%)	7 (3.5)	4 (6.2)	0.320	0.571
Heart Failure (%)	5 (2.5)	1 (1.5)	0.000	1.000
Hyperlipidemia (%)	67 (33.7)	31 (47.7)	4.128	0.042
Alcohol Consumption (%)	39 (19.6)	10 (15.4)	0.575	0.448
Smoking (%)	82 (41.2)	24 (36.9)	0.374	0.541
Vital signs				
Heart Rate (beats/min)	78.44 ± 12.74	75.00 ± 11.95	1.916	0.056
Systolic Blood Pressure (mmHg)	156.04 ± 23.98	153.94 ± 21.23	0.629	0.530
Diastolic Blood Pressure (mmHg)	90.49 ± 14.98	91.35 ± 14.57	−0.408	0.684
Laboratory parameters				
Glu (mmol/L)	6.37 (5.30,9.99)	6.10 (5.14,7.69)	−1.398	0.162
CYSC (mg/L)	0.87 (0.74,1.02)	0.81 (0.71,0.98)	−1.07	0.284
TC (mmol/L)	5.19 ± 1.35	5.13 ± 1.29	0.328	0.743
TG (mmol/L)	1.30 (1.06,1.81)	1.49 (0.96,2.03)	−0.961	0.337
HDL-C (mmol/L)	1.10 (0.93,1.33)	1.07 (0.96,1.32)	−0.027	0.978
LDL-C (mmol/L)	3.33 (2.66,3.88)	3.19 (2.58,3.82)	−0.594	0.552
Lp (a) (mmol/L)	145.58 (84.38,275.89)	127.07 (89.66,221.31)	−0.529	0.597
Hcy (mmol/L)	13.95 (11.52,16.69)	13.53 (11.41,19.05)	−0.374	0.708
HbA1C (%)	6.65 (6.12,9.09)	6.15 (5.84,7.95)	−2.657	0.008
Clinical and imaging features				
Onset Time (hours)	29.00 (23.00,46.00)	29.00 (21.00,42.00)	−0.081	0.935
Infarction Location (%)				
Anterior Circulation	135 (67.8)	48 (73.8)	0.831	0.362
Posterior Circulation	64 (32.2)	17 (26.2)		
TOAST Classification (%)				
LAA	181 (91.0)	57 (87.7)	4.653	0.155
SAO	3 (1.5)	4 (6.2)		
ODC	8 (4.0)	1 (1.5)		
UND	7 (3.5)	3 (4.6)		
Vascular Stenosis (%)				
No Stenosis	74 (37.2)	26 (40.0)	−0.505	0.614
Single	83 (41.7)	27 (41.5)		
Multiple	42 (21.1)	12 (18.5)		

Glu, Blood glucose; CYSC, Cystatin C; TC, Total cholesterol; TG, Triglycerides; HDL_C, High density lipoprotein cholesterol; LDL_C, Low density lipoprotein cholesterol; LP(a), Lipoprotein a; Hcy, Homocysteine; HbA1C, Glycosylated hemoglobin; TOAST, Trial of Org 10,172 in Acute Stroke Treatment; LAA, Large-artery atherosclerosis; SAO, Small-artery occlusion; ODC, Stroke of other determined cause; UND, Stroke of undetermined cause.

### Primary outcomes

**Figure 2:**
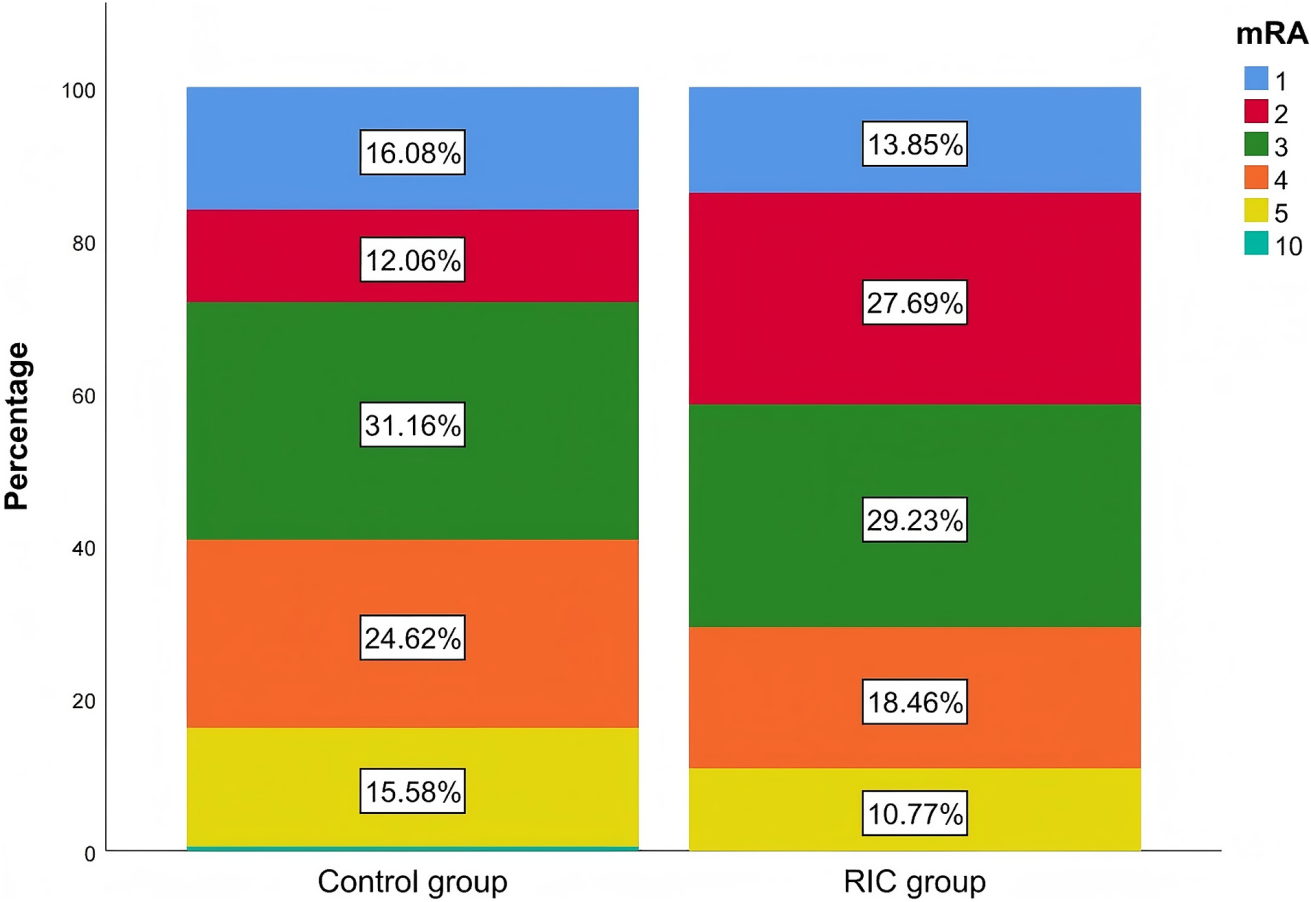
Proportions of mRS grade distributions in the two groups.

After 7 days, the RIC group had a significantly higher proportion of patients with an mRS score of ≤2 compared to the control group (41.5% vs. 28.1%, *p* = 0.043; Table 2). The Mann–Whitney U test demonstrated a statistically significant difference in mRS grade shifts from baseline between the two treatment groups (*p* < 0.05), and the stacked bar chart showed that the proportion of patients achieving mRS 0–2 after treatment was markedly higher in the RIC group than in the control group (Table 3; Figure 2). No recurrent strokes occurred in either group during the first week of follow-up.

**Table 2:** Comparison of evaluation indicators between RIC group and control group.

Variable	Group	Day 0	Day 7
NIHSS score	RIC group	4.00 (2.00,7.00)	2.00 (1.00,4.00)*△
	Control group	4.00 (3.00,7.00)	4.00 (2.00,6.00)*
mRS score≤2	RIC group	22 (33.8%)	27 (41.5%)*△
	Control group	54 (27.1%)	56 (28.1%)
ADL score	RIC group	60.00 (35.00,77.50)	65.00 (40.00,80.00)*△
	Control group	50.00 (25.00,65.00)	50.00 (25.00,65.00)
RLA score	RIC group	8.00 (7.00,8.00)	8.00 (7.50,8.00)
	Control group	8.00 (7.00,8.00)	8.00 (7.00,8.00)
D-Dimer	RIC group	769.26 ± 825.25	883.89 ± 865.52
	Control group	925.00 ± 1498.68	969.63 ± 1426.46
PT	RIC group	10.90 (10.40,11.40)	10.70 (10.15,11.05)*
	Control group	10.90 (10.40,11.40)	10.70 (10.20,11.10)*
APTT	RIC group	26.70 (23.85,27.95)	26.80 (25.20,28.10)
	Control group	27.00 (25.20,28.70)	27.20 (25.70,28.90)
Fibrinogen	RIC group	3.00 (2.66,3.43)	3.48 (3.06,4.02)
	Control group	3.32 (2.94,3.81)	3.85 (3.11,4.99)
WBC	RIC group	8.02 (6.59,9.78)	7.49 (6.03,8.60)*
	Control group	8.52 (6.95,10.15)	7.48 (6.26,9.11)*
CRP	RIC group	4.68 (2.25,7.77)	5.34 (2.79,9.16)**△**
	Control group	4.81 (2.96,11.60)	9.00 (4.09,18.40)*

*p < 0.05, compare with day 0; △: *p* < 0.05, compare with control. RIC, Remote ischemic conditioning; NIHSS, National Institute of Health Stroke Scale; mRS, The Modified Rankin Scale; ADL, Activities of daily living; RLA, Rancho Los Amigos; PT, Prothrombin time; APTT, Activated partial thromboplastin time; WBC, White blood cell; CRP, C-reactive protein.

**Table 3:** Between-group differences in mRS grade distributions.

Variable	Control group	RIC group	Z/*χ^2^*	*p*-value
mRS(day 0)[*M* (*P_25_*, *P_75_*)]	3 (2,4)	3 (2,4)	0.537	0.591
mRS(day 7)[*M* (*P_25_*, *P_75_*)]	3 (2,4)	3 (2,4)	1.712	0.087
Change from baseline of mRS[*M* (*P_25_*, *P_75_*)]	0 (0,0)	0 (0,0)	2.464	0.014
mRS(day 0) [n,(%)]			1.076	0.300
≤2	54 (27.14)	22 (33.85)		
>2	145 (72.86)	43 (66.15)		
mRS(day 7)[n,(%)]			4.080	0.043
≤2	56 (28.14)	27 (41.54)		
>2	143 (71.86)	38 (58.46)		

mRS, The Modified Rankin Scale.

### Secondary outcomes

There were no significant differences in baseline NIHSS, ADL, and RLA scores between the two groups (*p* > 0.05). After 7 days, the RIC group showed a significant reduction in NIHSS scores and an increase in ADL scores compared to baseline (*p* < 0.05). These differences were also statistically significant compared to the control group (*p* < 0.05). Covariance analysis, adjusting for baseline differences (e.g., hyperlipidemia and HbA1C), confirmed that the differences in NIHSS and ADL scores after 7 days of treatment remained significant (FNIHSS = 8.406, PNIHSS = 0.004; FADL = 8.244, PADL = 0.005). However, there was no significant difference in RLA scores before and after the intervention, or between the two groups.

### Laboratory measures

Analysis of coagulation function (including PT, APTT, D-Dimer and Fibrinogen) and inflammation markers (WBC and CRP) revealed no intergroup differences in coagulation indicators at baseline or after 7 days of treatment. However, CRP levels significantly increased in the control group compared to the RIC group after 7 days of treatment (*p* = 0.001), and WBC levels showed a significant decrease post-treatment, though intergroup differences were not statistically significant.

### Binary logistic regression

To explore the effects of different treatment methods on the outcome, both unadjusted and adjusted for variables. In Model 1 (unadjusted), the odds ratio (OR) for achieving a favorable prognosis (mRS ≤ 2) in the RIC group was 0.55 (95% CI: 0.31–0.99, *p* = 0.045), compared to the control group. In Model 2, which adjusted for age, sex, and baseline mRS scores (day 0), the OR for the RIC group was 0.30 (95% CI: 0.12–0.78, *p* = 0.014). In Model 3, which further adjusted for hyperlipidemia and HbA1C, the OR for the RIC group was 0.28 (95% CI: 0.11–0.78, *p* = 0.014; Table 4).

**Table 4:** Binary logistic regression analysis of different treatment and patient prognosis.

Variable	OR (95%CI)	*p*-value
Model 1		
Control group	Ref	
RIC group	0.55 (0.31,0.99)	0.045
Model 2		
Control group	Ref	
RIC group	0.30 (0.12,0.78)	0.014
Model 3		
Control group	Ref	
RIC group	0.28 (0.11,0.78)	0.014

Model 1: unadjusted model. Model 2: adjusted for age, sex, mRS(day 0). Model 3: adjusted for age, sex, mRS (day 0), Hyperlipidemia, HbA1C. RIC, RemoteIschemic Conditioning; OR, Odds ratio.

## Discussion

In this study, we aimed to evaluate the efficacy of RIC in improving neurological function and short-term prognosis in patients with AIS. The results indicate that RIC treatment can significantly improve functional outcomes, with a higher proportion of patients achieving an mRS score of ≤2 at day 7 and improvements in NIHSS and ADL scores. Importantly, logistic regression analysis confirmed that even after adjusting for baseline variables, patients in the RIC group had significantly higher odds of achieving a favorable short-term prognosis than those in the control group. These findings suggest that RIC holds promise as an adjunctive therapy for AIS patients.

Despite significant advancements in reperfusion therapies for AIS, including intravenous thrombolysis and mechanical thrombectomy, clinical evidence shows that not all patients benefit clinically from successful recanalization (15, 16). This phenomenon, often termed “futile recanalization,” highlights the need for adjunctive strategies beyond vessel reopening (17, 18). Neuroprotection and neurorepair approaches, such as RIC, may offer additional benefits by targeting the cascade of cellular and molecular events that continue after reperfusion is achieved (5, 6, 19). This provides a strong rationale for exploring RIC as a complementary therapy, particularly for patients who may not fully respond to conventional reperfusion treatments or those who fall outside the narrow therapeutic windows for these interventions (2–4).

RIC’s mechanisms extend beyond acute neuroprotection to encompass potential neurorepair processes (20, 21). While early application of RIC primarily provides neuroprotection through reduction of oxidative stress and suppression of inflammation (7, 8), repeated RIC post-conditioning (RIpostC) as applied in our protocol may activate longer-term neurorepair mechanisms (22). These include enhancement of cerebral collateral circulation (23, 24), promotion of angiogenesis (25), stimulation of endogenous neurogenesis (26), and modulation of neural plasticity (27). The repeated application of RIC over 7 days, as implemented in our study, may be particularly effective in initiating these repair processes that extend beyond the acute phase of stroke (13, 28). This dual action of protection and repair could explain the significant improvements observed in functional outcomes in our RIC group compared to controls.

Studies by Kolpakova et al. showed that RIC treatment significantly reduced infarct size and improved neurological scores in rats (29). Clinical research, such as the RECAST study, found that RIC is safe and feasible for AIS patients within 24 h of onset, with significantly lower NIHSS scores at 90 days in the RIC treatment group compared to the sham RIC group [1.0 (0.55.0) vs. 3.0 (2.09.5), *p* = 0.04] (13). Another study involving 60 AIS patients within 72 h of onset found that RIC significantly improved cerebral perfusion in the ischemic area and reduced 90-day NIHSS scores, with a 31.3% reduction in final infarct volume (30). In addition, the recently published REMOTE-CAT trial investigated the effects of prehospital RIC in acute stroke patients and found that RIC initiated in the ambulance setting did not significantly improve clinical outcomes at 90 days compared to standard care (31). However, the authors noted challenges such as protocol adherence and patient selection, which may have limited the efficacy signal. Our study observed significant effects of RIC in a shorter 7-day follow-up, suggesting that early short-term RIC treatment (7 days) can effectively improve neurological function prognosis in AIS patients.

Compared to existing literature, this study is the first to comprehensively evaluate the impact of RIC on neurological function and daily living abilities in AIS patients. The findings show that patients in the intervention group had significantly better outcomes across multiple functional assessment indicators after RIC treatment compared to the control group. This suggests that RIC can improve short-term neurological function, and significantly enhance daily living abilities in a short period. Additionally, there was no improvement in RAL scores before and after treatment, suggesting that RIC therapy does not significantly enhance cognitive function post-stroke. This finding is consistent with the RECAST study (13).

Our study incorporated a shift analysis, which provides a robust evaluation of the changes in mRS scores from baseline to day 7. This analysis confirmed that the RIC group showed significantly greater improvements compared to the control group, reinforcing the observed benefits of RIC treatment. We also binary logistic regression to explore the effects of different treatment methods on the outcome. After adjusting for baseline factors, including age, sex, and baseline mRS scores (Model 2), as well as additional factors such as hyperlipidemia and HbA1C levels (Model 3), the treatment effect of RIC remained statistically significant. This indicates that the observed benefits of RIC were not confounded by baseline differences, further supporting the robustness of our findings.

Regarding RIC operational parameters, some studies have found that different ischemia times and cycle numbers affect RIC efficacy (32, 33). Future research should further explore the optimal RIC operational parameters. Common clinical studies often use four-cycle RIC treatment (13, 30, 34) or five-cycle RIC intervention methods (35–38). Most studies apply bilateral or unilateral upper limb RIC treatments (39). Our study adopted a five-cycle bilateral limb RIC standard operation scheme, demonstrating significant clinical effects and indicating its feasibility in clinical application. Notably, the RESCUE-BRAIN study (34), one of the few studies not supporting the efficacy of RIC for AIS, also used a rare single lower limb RIC ischemia intervention with four cycles. Whether limb selection and the number of ischemia cycles contributed to the different results warrants further investigation in future studies.

This study has several strengths. The 1:3 group allocation enhances statistical power, and the comprehensive outcome measures, including NIHSS, mRS, ADL, and RLA scores, provide a thorough evaluation of neurological function and daily living abilities. Additionally, the inclusion of inflammation and coagulation markers offers valuable insights into the biological mechanisms of RIC. Furthermore, the robust sample size and high retention rate increase the reliability of the findings. However, this study also has certain limitations. As a single-center study with a relatively short follow-up period, the generalizability and long-term effects of RIC require further investigation. Despite baseline factors adjustment, baseline differences in hyperlipidemia and HbA1c may reflect residual confounding. Larger trials with stratified randomization are needed to confirm the generalizability of our findings. Another important limitation of this study is the absence of neuroimaging outcome measures, particularly assessment of infarct volume. Quantitative evaluation of infarct volume through follow-up MRI would have provided crucial information on the potential tissue-salvaging effects of RIC and could have helped elucidate the mechanisms underlying the observed clinical improvements. Furthermore, while our 7-day assessment provides valuable insights into the immediate effects of RIC during a critical period of early recovery, it does not allow us to determine whether these benefits are sustained over time or translate to long-term functional improvements. Future studies should involve larger sample sizes, multicenter trials, and extended follow-up periods to fully assess the clinical efficacy and safety of RIC in AIS patients. Additionally, we did not collect data on oxidative stress markers such as NLR, SOD, MDA, or oxLDL, which could provide valuable insights into the underlying mechanisms of RIC in reducing oxidative damage and inflammation. Future research should incorporate these markers to further explore the broader physiological impacts of RIC.

## Conclusion

RIC, as a non-invasive and low-cost treatment, shows significant potential in improving neurological function and daily living abilities in AIS patients. The findings of this study provide new evidence supporting the clinical application of RIC, especially for patients who cannot undergo conventional reperfusion therapies. RIC offers an effective alternative treatment option.

## Data Availability

The original contributions presented in the study are included in the article/Supplementary material, further inquiries can be directed to the corresponding author.
