# Supplementary material for: The efficacy of remote ischemic conditioning in improving neurological function and short-term prognosis in acute ischemic stroke: a prospective controlled study
Source: Front Neurol. 2025 Jul 1;16:1542833. doi: 10.3389/fneur.2025.1542833 (PMC12259425; doi:10.3389/fneur.2025.1542833)
Supplement: Supplementary file 1 [file Table_1.docx]

Supplementary Table 1. Linear Regression of Post-Treatment mRS Scores by Treatment Modality

|  | Coef(95%CI) | p-value | **Adjusted R square** |
| --- | --- | --- | --- |
| Model 1 |  |  |  |
| Control group | Ref |  | 0.006 |
| RIC group | -0.305(-0.678,0.069) | 0.110 |  |
| Model 2 |  |  | 0.745 |
| Control group | Ref |  |  |
| RIC group | -0.219(-0.409,-0.029) | 0.024 |  |
| Model 3 |  |  | 0.744 |
| Control group | Ref |  |  |
| RIC group | -0.208(-0.402,-0.015) | 0.035 |  |

Model 1: unadjusted model.

Model 2: adjusted for age,sex,mRS(day 0)

Model 3: adjusted for age,sex,mRS(day 0),Hyperlipidemia,HbA1C

we performed a linear regression analysis using mRS (day 7) as the dependent variable and different treatment modalities (among other factors) as independent variables to examine the effect of each treatment modality on mRS (day 7) both before and after adjusting for baseline covariates. The results showed that in the unadjusted (crude) model, the effect estimate for the RIC group relative to the Control group was not statistically significant. However, after adjusting for baseline variables, the effect estimate for the RIC group versus the Control group was below 1 and reached statistical significance (P < 0.05). This indicates that baseline mRS may have masked the relationship between treatment modality and outcome score, and that adjusting for baseline level reveals the independent effect of the treatment modality.
